# Seven-Compartment Fascial Release of the Lower Extremity: A Case Report

**DOI:** 10.7759/cureus.32023

**Published:** 2022-11-29

**Authors:** Blake Callahan, Darwin Ang

**Affiliations:** 1 Department of Surgery, University of Central Florida College of Medicine, Ocala, USA

**Keywords:** seven compartment fasciotomy, wound closure, lower extremity trauma, medial thigh, compartment syndrome, fasciotomy

## Abstract

Compartment syndrome of the lower extremities is a condition that can lead to permanent nerve and muscle damage if not treated in an emergent fashion. Fasciotomy of the medial compartment of the thigh is exceedingly rare, and a review of the literature revealed only one reported case where compartment syndrome was present in both the thigh and lower leg compartments simultaneously. Given the rarity of compartment syndrome in all seven compartments of the leg, in this case, we report the development of full leg compartment syndrome in a 29-year-old male who fell asleep on a hard surface for an extended period following heroin intoxication, which was treated with seven compartment fasciotomies. We conclude with a discussion about how medial release of the thigh for compartment syndrome is rare enough that careful consideration of the anatomy must be made before proceeding with the procedure. Additionally, wound closure has many proposed options, but current literature favors skin staples with an interlaced elastic band to minimize delays in wound closure.

## Introduction

In the thigh, there are three primary compartments: the anterior compartment, which includes the sartorius and quadriceps muscles; the posterior compartment, which includes the hamstring muscles; and the medial compartment, which is comprised of the adductor muscles. In the lower leg, there are four compartments comprised of the anterior, lateral, superficial, and deep posterior compartments. The tibialis anterior, extensor muscles of the foot, and fibularis tertius muscles are located in the anterior compartment. In the lateral compartment, there are the fibularis longus and brevis muscles. The gastrocnemius, soleus, and plantaris muscles are located in the superficial posterior compartment. In the deep posterior compartment, the tibialis posterior, flexor muscles of the foot, and popliteus muscles can be found.

Compartment syndrome of the lower extremities is a surgical emergency requiring immediate fasciotomy and release of the fascia enveloping the muscular compartments. There are seven compartments that comprise the thigh and lower leg. Compartment syndrome can occur in any compartment, often following blunt or sharp injuries, overuse, or even spontaneously. Compartment syndrome of the lower leg is far more common than compartment syndrome of the thigh [1], and a review of the literature revealed only one reported case where compartment syndrome was present in both the thigh and lower leg compartments simultaneously [2].

Given the rarity of compartment syndrome in all seven compartments of the leg, in this case, we report the development of full-leg compartment syndrome in a 29-year-old male who fell asleep on a hard surface for an extended period following heroin intoxication. This was treated with seven-compartment fasciotomies.

The lower extremity, excluding the ankle and foot, is comprised of seven distinct compartments housing various muscle groups and contained within the fascia and the muscular septum. Increased pressure in these relatively fixed spaces is caused by any number of conditions and can lead to compression and ischemia of vital neuromuscular structures, possibly leading to muscle necrosis and nerve injury. This clinical scenario is called compartment syndrome and is deemed a surgical emergency. A surgical procedure to incise the fascial compartments must be done within a window of time reported as being three to 12 hours after the onset of symptoms before possibly permanent structural damage occurs [3].

## Case presentation

A 29-year-old male with a past medical history of heroin use presented to the emergency department with pain, numbness, and weakness of the bilateral lower extremities after falling asleep for an extended period after using intravenous heroin.

The patient’s physical exam on presentation was as follows: The patient was alert and oriented, well developed and nourished, and in no apparent distress, but tachycardia was noted. The patient was unable to move the right lower extremity and had an absence of sensation over the tibial, sural, saphenous, superficial peroneal, and deep peroneal nerve distributions. His medial thigh compartment was full and tense to inspection and palpation. The right lower leg compartments were full and tense to inspection and palpation. The absence of sensation to light touch was noted up to the groin. His right dorsalis pedis pulse was decreased in intensity (1+) in comparison to the contralateral side (2+). The right lower extremity was cool to the touch.

**Figure 1:**
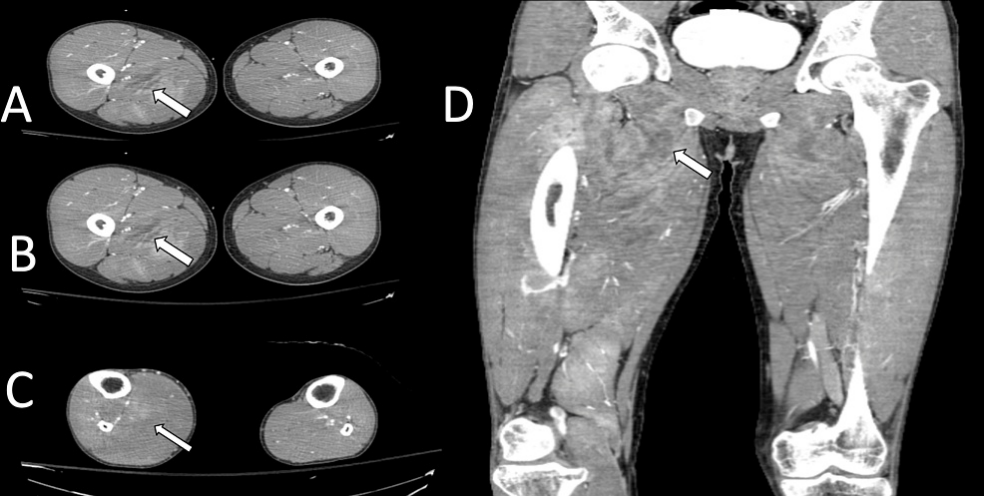
Axial and coronal computed tomography images of the lower extremities Axial and coronal computed tomography images of the lower extremities show extensive and diffuse myositis (hypointense signal) (axial A–C, coronal D)

Computer tomography (CT) scans of the lower extremities showed a diffuse abnormal appearance with hyper- and hypodensity as well as enhancement involving the extensor and flexor compartments of the thigh. Edema with an abnormal appearance of the right calf musculature was present. These findings were characteristic of extensive diffuse myositis. Axial images of these findings are shown in Figure 1.

Notable lab values included a markedly elevated white blood cell count of 33.4; his hemoglobin was 17.9 g/dL; his hematocrit was 53.1%. He had electrolyte derangement, with a critical potassium level of 7.3 meq/dL. His creatinine was 1.6 mg/dL. Creatinine phosphokinase (CPK) was measured at 28,219 ng/mL. Troponin I levels were elevated to 0.484 ng/ml (normal is less than 0.04 ng/ml) and lactic acidosis of 10.6 mmol/L was recorded.

The patient was taken to the operating room for emergent fasciotomies. Beginning in the lower leg compartments, we incised the lateral and anterior compartments, where bulging muscles were encountered. The tissue appeared viable and red but only minimally reacted to electrostimulation. We then continued to the superficial and deep posterior compartments, where the muscle in the deep posterior compartment was noted to have areas of dark red ecchymoses; however, it did contract to electrostimulation.

**Figure 2:**
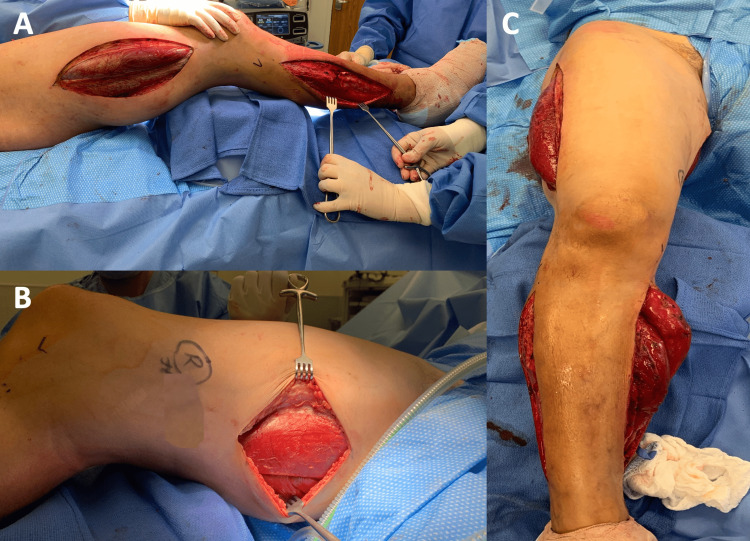
Photographs of the seven completed compartment fasciotomies (A) A side view of the upper and lower leg fasciotomy incisions, demonstrating bulging and edematous musculature. (B) A side view of the completed fasciotomy incision of the thigh's medial compartment, demonstrating bulging and edematous musculature. (C) A frontal view of the completed fasciotomies with protruding muscles of the leg compartments.

After fasciotomies of the lower leg, we moved on to perform fasciotomies of the thigh compartments. We began with a lateral thigh incision to release the anterior and posterior compartments of the thigh. The vastus lateralis muscle was noted to react well to stimulation. Biceps femoris appeared viable but only minimally responded to stimulation. Given the significantly tight medial/adductor compartment of the thigh, we opted to make an incision over the medial aspect of the thigh, and we released the medial compartment as well as the posterior compartment from the medial aspect. Adductor muscles appeared viable and red but minimally responded to electrostimulation. The patient tolerated the procedure well and was sent to recover in the intensive care unit for postoperative observation. Photographs of the seven completed compartment fasciotomies are shown in Figure 2.

Four days later, the patient underwent right lower extremity fasciotomy site washout and partial closure of all compartments except for the medial lower leg compartments. Four days after partial closure, the final incision on the medial aspect of the lower leg was washed out, and the application of a wound vacuum device was applied to the remaining open wound. On hospital day 14, the patient had a split-thickness skin graft placed and was able to walk on his affected leg using crutches with full sensation and the ability to move his foot and ankle.

## Discussion

This case of acute compartment syndrome is a rare one, as it involved all seven leg compartments while also including an incision along the medial aspect of the thigh. When discussing thigh compartment syndrome, for most presentations, a reported 88% are treated with a single lateral incision [1]. When a second medial incision has been left unutilized, there is little data regarding the outcomes of such measures. There are several anatomical factors to consider when conducting a medial thigh fasciotomy. Superficially, the saphenous vein and nerve run medially, as with the superior geniculate arteries of the distal femur. Upon deeper dissection, there is the neurovascular bundle of the adductor canal and obturator nerve, which innervates the adductor muscles of the medial thigh. Careful consideration should be given to avoiding damage to these structures to prevent bleeding and neurological injury.

Another notable characteristic of this case is the staged, delayed closure of the fasciotomy incisions. For the thigh, wound closure recommendations are unclear. One retrospective study found that primary closure was performed in 22.2% of thigh compartment syndrome cases, delayed primary closure in 11.1%, split-thickness skin grafting in 33%, full-thickness skin grafting in 11.1%, and above-the-knee amputation with split-thickness skin grafting in 11.1% of thigh fasciotomy cases [4]. One paper demonstrated a shoelace technique where staples are interwoven with an interlaced elastic band as a choice for delayed wound closure [5]. Another popular technique is delayed wound closure with the application of wound vacuum devices. One randomized trial suggested that closure time was significantly longer when a wound vacuum was used in comparison to a group utilizing the shoelace technique, with the study finding that a wound vacuum could add up to 6.8 days to the closure [6].

## Conclusions

Seven-compartment fasciotomies are rare but may be indicated in severe cases of myositis or myonecrosis from trauma. Early identification and surgical intervention may not only prevent mortality but also preserve function. The medial compartment of the thigh may also need to be released, as it is also at risk for compartment syndrome. This report is the first to describe a seven-compartment release in a civilian setting.

When performing a medial release of the thigh for compartment syndrome, careful consideration of the anatomy must be made before proceeding with the procedure. Wound closure has many proposed options, but the current literature favors skin staples with an interlaced elastic band to minimize delays in wound closure.
